# Epitranscriptomic Regulation of Platinum Resistance via the METTL3-ADAM23 Axis in Ovarian Cancer

**DOI:** 10.3390/cells15030294

**Published:** 2026-02-04

**Authors:** Ujin Kim, Junzui Li, Daniela Matei, Hao Huang

**Affiliations:** 1 Department of Obstetrics and Gynecology, Feinberg School of Medicine, Northwestern University, Chicago, IL 60611, USA; 2 Robert H. Lurie Comprehensive Cancer Center, Feinberg School of Medicine, Northwestern University, Chicago, IL 60611, USA; 3 Jesse Brown VA Medical Center, Chicago, IL 60612, USA

**Keywords:** ovarian cancer, epitranscriptomic, RNA methylation, platinum resistance, METTL3, ADAM23

## Abstract

**Highlights:**

**What are the main findings?**

- METTL3 expression and global m^6^A RNA methylation are increased following platinum treatment and in platinum-resistant ovarian cancer models.
- ADAM23 is identified as a METTL3-regulated, m^6^A-associated transcript whose expression correlates with platinum response.

**What is the implication of the main finding?**

- METTL3-dependent epitranscriptomic regulation is associated with the development of platinum resistance in ovarian cancer.
- Pharmacologic inhibition of METTL3 enhances platinum responsiveness, supporting m^6^A regulation as a potential therapeutic vulnerability.

**Abstract:**

N6-methyladenosine (m^6^A) has emerged as a pivotal regulator of post-transcriptional gene control, yet its contribution to chemotherapy resistance remains insufficiently defined. Here, we describe a previously unrecognized METTL3-ADAM23 epitranscriptomic regulatory relationship associated with platinum (Pt) resistance in ovarian cancer (OC). We show that cisplatin treatment increases global m^6^A levels and METTL3 expression, linking Pt exposure to activation of the m^6^A machinery. Functional perturbation studies demonstrate that METTL3 overexpression enhances cisplatin resistance, whereas METTL3 knockdown or pharmacologic inhibition with the selective METTL3 inhibitor STM2457 sensitizes OC cells to Pt treatment in vitro and improves Pt response in vivo. Transcriptomic profiling identifies ADAM23, a cell-adhesion-related tumor suppressor, as a METTL3-dependent, m^6^A-associated transcript, with altered mRNA expression observed across multiple experimental systems and several high-confidence predicted m^6^A sites within its transcript. Cisplatin-associated METTL3 upregulation correlates with reduced ADAM23 expression, suggesting a potential regulatory relationship that may contribute to chemoresistance. Together, these findings support a model in which METTL3-associated increases in m^6^A methylation are linked to Pt resistance, in part through modulation of ADAM23 expression, and highlight METTL3 as a potential therapeutic target in OC.

## 1. Introduction

Ovarian cancer (OC) remains a leading cause of cancer death among women. Even with newly approved treatments, the five-year survival rate is still less than 50%. The main reasons for the low survival rate are late diagnosis and chemoresistance. Platinum (Pt)-based chemotherapy, primarily using carboplatin or cisplatin, is the cornerstone of first-line treatment for OC. While initial response rates are high, most patients subsequently relapse and develop resistance to Pt agents. Additionally, up to 15–30% of patients experience primary Pt resistance [1,2]. Treatment for chemo-resistant tumors is limited and includes second-line agents, targeted therapy and antibody drug conjugates [3,4,5]. Consequently, deciphering molecular drivers of Pt resistance is essential to developing new, more effective therapies [6].

Epitranscriptomic modifications refer to chemical modifications of RNA molecules after transcription and represent a key mechanism that controls gene expression in cancer [7,8]. N6-methyladenosine (m^6^A) is the most common RNA modification and affects gene regulation by altering RNA structure, stability, localization, and processing, leading to alterations in splicing, translation, and mRNA degradation [9]. Through these effects, m^6^A modifications influence key biological processes, including cancer progression and response to treatment [10]. M^6^A modifications are deposited by methyltransferase “writer” complexes, removed by demethylase “erasers,” and recognized by m^6^A-binding “reader” proteins. The catalytic core of the writer complex consists of METTL3 and METTL14, which work with cofactors including WTAP, VIRMA, and RBM15 to target methylation at RRACH consensus sequences in mRNA [11,12]. METTL3 promotes tumorigenesis across diverse cancers, such as acute myeloid leukemia, where it promotes leukemic cells’ survival by enhancing MYC/BCL2 translation [13] and hepatocellular, lung, and breast cancers, where it stabilizes oncogenic mRNAs to drive proliferation and metastasis [14,15,16]. Increased expression of METTL3 in OC is linked to aggressiveness, epithelial to mesenchymal transition (EMT), invasion, and poor survival [17,18,19]. However, its involvement in drug resistance remains undefined.

In this study, we hypothesized that METTL3 may contribute to Pt resistance through epitranscriptomic regulation of the tumor-suppressive pathway. Using integrative molecular and transcriptomic analyses, we identified disintegrin and metalloprotease domain-containing protein 23 (ADAM23) as a METTL3-regulated, m^6^A-associated transcript linked to Pt resistance. ADAM23 belongs to the ADAM family of transmembrane metalloproteases, involved in cell adhesion, extracellular matrix remodeling, and signal transduction [20]. Previous studies have reported that ADAM23 functions as a tumor suppressor and is frequently silenced in breast, pancreatic, and gastric cancers through promoter hypermethylation [21,22,23]. However, the potential involvement of ADAM23 in OC and its relationship to Pt response have not been previously defined.

Here, we show that METTL3 and global m^6^A levels are increased following cisplatin treatment in OC cells. METTL3 forced expression enhanced platinum resistance, whereas METTL3 knockdown (KD) or pharmacologic inhibition with the selective METTL3 inhibitor STM2457 increased cisplatin sensitivity. Our data are consistent with a model in which the METTL3-dependent, m6A-associated regulation of ADAM23 mRNA is linked to reduced transcript abundance, thereby influencing cellular responsiveness to Pt. Collectively, these findings propose a new METTL3–ADAM23 regulatory relationship that may contribute to Pt resistance. These observations provide insight into how m^6^A-associated regulatory programs shape therapeutic response and highlight new potential targets for improving treatment outcomes in OC.

## 2. Materials and Methods

### 2.1. Cell Culture

OVCAR4 and OVCAR5 cells were kindly provided by Dr. Mazhar Adli and Dr. Marcus Peter (Northwestern University, Evanston, IL, USA), respectively. COV362 cells were provided by Dr. Kenneth Nephew (Indiana University, Bloomington, IN, USA). OVCAR4 cells were cultured in RPMI-1640 supplemented with 10% fetal bovine serum (FBS) and 1% penicillin–streptomycin. OVCAR5 cells were maintained in RPMI-1640 with L-glutamine (Corning, Cat# 10-040CV, Corning, NY, USA), supplemented with 10% FBS, 1% GlutaMAX, and 1% penicillin–streptomycin. COV362 cells were cultured in high-glucose DMEM supplemented with 10% FBS, 1% penicillin–streptomycin, and 1× GlutaMAX. HEK293T cells were grown in DMEM supplemented with 10% FBS and 1% penicillin–streptomycin.

Murine ID8 OC cells were provided by Dr Katherine Roby (University of Kansas, Lawrence, KS, USA). ID8-luc were generated by transducing ID8 cells with pLX311 Luciferase (Luc) and selected by blasticidin. ID8 and ID8-luc cells were grown in DMEM, supplemented with 10% fetal bovine serum (FBS) and 1% penicillin/streptomycin solution.

Cell lines were confirmed to be free of pathogens and mycoplasma by Charles River Research Animal Diagnostic Services (Wilmington, MA, USA) and were periodically tested using the MycoAlert^®^ Mycoplasma Detection Kit (Lonza, Cat# LT07-418, Cambridge, MA, USA). Experiments were performed using low-passage cells.

### 2.2. Chemicals and Reagents

Cisplatin (cat. # 1134357) and carboplatin (cat. # C2538) were purchased from Sigma-Aldrich (St. Louis, MO, USA). STM2457 was from MedchemExpress (MCE, cat. # HY-134836, Monmouth Junction, NJ, USA), and D-Luciferin was from GoldBio (cat. # LUCK-100, St. Louis, MO, USA).

### 2.3. Plasmid Construction

Lentiviral METTL3 pCDH expression vectors and the lentiviral shRNA constructs targeting METTL3 (designated shMETTL3-1 and shMETTL3-2) were generously supplied by Dr. Jianjun Chen at City of Hope. The shMETTL3 constructs correspond to MISSION shRNAs from Sigma-Aldrich with the following clone IDs and target sequences: TRCN0000034717(shMETTL3-1; target sequence: 5′-AACAGCCAAGGAACAATCCATTGTT-3′) and TRCN0000289814 (shMETTL3-2; target sequence: 5′-AAGCTGCACTTCAGACGAATTATC-3′).

Lentiviral vectors encoding human ADAM23 shRNA and the corresponding non-targeting control were obtained from Applied Biological Materials (Richmond, BC, Canada). Lentiviral particles were generated in 293T cells by co-transfecting the expression vectors with the packaging plasmids VSV-G and psPAX2. Viral supernatants were collected 72 h post-transfection and used to infect target cells in the presence of polybrene (8 µg/mL) for 48 h.

### 2.4. Cell Survival Assay

Cells were exposed to increasing concentrations of platinum (Pt) for 24 h, after which the drug-containing medium was replaced and cultures were maintained for an additional 72 h. Cell viability was then assessed using the Cell Counting Kit-8 assay (CCK-8; Dojindo Molecular Technologies, Rockville, MD, USA) according to the manufacturer’s instructions. Absorbance at 450 nm was recorded using a BioTek ELX800 microplate reader (BioTek, Winooski, VT, USA).

### 2.5. RNA Extraction and Quantitative RT-PCR Analysis

RNA was isolated with TRIzol reagent (Invitrogen, Carlsbad, CA, USA) and quantified spectrophotometrically. Reverse transcription was performed using 1 μg RNA and the iScript cDNA synthesis system (Bio-Rad). Quantitative PCR analysis was conducted with SYBR Green chemistry (iTaq Universal SYBR Green Supermix, Bio-Rad, Hercules, CA, USA) on an ABI 7900HT platform.

Expression values were normalized to 18S rRNA, and relative abundance was determined using the ΔΔCt approach. Primer information is listed in Supplementary Table S1. All reactions were performed in triplicate, and results represent the mean ± SD of independent experiments.

### 2.6. Western Blotting

Whole-cell lysates were obtained in RIPA buffer, followed by sonication and clarification by centrifugation. Protein concentrations were determined by using the Bradford method (Bio-Rad, Hercules, CA, USA). Equal amounts of protein were separated by SDS–PAGE and transferred onto PVDF membranes. After blocking with 5% skim milk, membranes were incubated overnight at 4 °C with primary antibodies against METTL3 (Abcam, Cambridge, UK, Cat. # ab195352) or GAPDH (Thermo Fisher Scientific, Waltham, MA, USA, Cat. #AM4300), followed by incubation with appropriate secondary antibodies. Immunoreactive signals were visualized by using enhanced chemiluminescence (SuperSignal™ West Pico PLUS, Thermo Fisher Scientific, Waltham, MA, USA).

### 2.7. m^6^A Dot Blot Assay

mRNA was purified from total RNA using the Dynabeads™ mRNA Purification Kit (Ambion, Austin, TX, USA) and quantified with a NanoDrop 2000 spectrophotometer. The m^6^A dot blot assay was conducted based on a previously published protocol (www.bio-protocol.org/e2095, 5 January 2017), with minor modifications. Briefly, purified mRNA was spotted onto Amersham Hybond-N+ membranes (GE Healthcare, Chicago, IL, USA) and immobilized by UV crosslinking. Membranes were blocked with 5% non-fat dry milk and incubated overnight at 4 °C with an anti-m^6^A antibody (Synaptic Systems, Goettingen, Germany.), followed by incubation with an HRP-conjugated goat anti-rabbit secondary antibody (Santa Cruz Biotechnology, Dallas, TX, USA) at room temperature for 1 h. Chemiluminescent signals were detected by using a substrate from Thermo Scientific prior to imaging.

### 2.8. Quantification of Global m^6^A RNA Methylation

Global m^6^A RNA methylation levels were quantified by using the EpiQuik™ m^6^A RNA Methylation Quantification Kit (Epigentek, Farmingdale, NY, USA). Briefly, 300 ng of total RNA per sample was processed through sequential antibody-based detection steps according to the assay workflow. Absorbance was measured at 450 nm using an xMark microplate reader (Bio-Rad, Hercules, CA, USA).

### 2.9. In Vivo ID8-Luciferase (Luc) Intraperitoneal Model

All animal procedures were approved by the Institutional Animal Care and Use Committee (IACUC) at Northwestern University (protocol # IS00026404) and conducted in accordance with institutional and NIH guidelines. Female C57BL/6 mice (6–8 weeks old) were obtained from Envigo (Indianapolis, IN, USA). Mice were injected intraperitoneally (ip) with 5 × 10^6^ murine ID8-luciferase (ID8-luc) cells to establish peritoneal tumors. Treatment began following tumor cell implantation. STM2457 (50 mg/kg) [24] or vehicle control was administered i.p. once daily for 16 consecutive days, starting 3 days after tumor inoculation (*n* = 5 mice per group). Carboplatin (40 mg/kg) was administered i.p. once weekly, beginning 7 days after tumor implantation. Tumor progression was monitored by bioluminescence imaging (Lago system) on day 3 and day 20 prior to euthanasia. The bioluminescent signal was quantified following intraperitoneal injection of D-luciferin (150 mg/kg), and images were acquired once a stable peak signal was reached. Total photon flux (photons/s) was calculated using identical exposure settings for all animals. Background-subtracted flux values were used to compare tumor burden across treatment groups. Mice were monitored daily and euthanized on day 24 or upon development of abdominal ascites, ≥15% body-weight gain or loss, body condition score ≤2, or signs of distress, including impaired mobility or marked lethargy, prior to the preset endpoint of the experiment.

### 2.10. RNA-Sequencing

RNA-seq libraries were generated from 1 μg of total RNA following mRNA enrichment using the NEBNext^®^ Ultra™ II RNA Library Prep Kit (New England Biolabs, Ipswich, MA, USA). Library construction included first- and second-strand cDNA synthesis, end repair and A-tailing, adaptor ligation, and PCR amplification. Library quality and size distribution were assessed using a High-Sensitivity DNA Bioanalyzer. Sequencing was performed on an Illumina NovaSeq platform to generate single-end 50 bp reads. Raw FASTQ files were processed with FastQC and aligned to the hg38 human genome using hisat2. Gene counts were generated using Subread and analyzed using DESeq2 to identify differentially expressed genes, as we described previously [25,26].

### 2.11. Bioinformatic and Public Dataset Analyses

Public proteomic data for METTL3 were obtained using the Clinical Proteomic Tumor Analysis Consortium (CPTAC) proteomics module of the University of Alabama at Birmingham CANcer (UALCAN) web portal (http://ualcan.path.uab.edu, 13 December 2021) [27]. METTL3 protein Z-scores derived from CPTAC OC samples were retrieved directly from UALCAN, which provides standardized spectral-count-based protein abundance across normal adjacent tissues and primary tumors. Bulk RNA-seq data associated with cisplatin sensitivity based on transcriptomic signature were retrieved from the Cancer Pharmacogenetics and Drug Sensitivity (CPADS, https://smuonco.shinyapps.io/CADSP/, 3 January 2023) [28] database. Cisplatin-response-related METTL3 expression was analyzed by using the Drug Analysis module of the CPADS database. Within CPADS, ovarian serous cystadenocarcinoma was selected as the cancer type, cisplatin as the drug of interest, and METTL3 as the query gene. CPADS automatically stratifies samples into IC50-high (resistant) and IC50-low (sensitive) groups based on transcriptomic signature and provides normalized METTL3 expression values together with the corresponding statistical comparison (two-group Wilcoxon test). The METTL3 expression difference and *p*-value reported by CPADS were used directly for downstream interpretation.

Single-cell RNA-seq datasets GSE184880 [29] and GSE222556 [30] were downloaded from GEO and processed using the same parameters described in the original publications to reproduce the reported preprocessing and clustering structure. After reproducing the authors’ pipeline, METTL3 expression was visualized on the UMAP embeddings of GSE184880 to examine its distribution across epithelial, stromal, endothelial, immune, and other cell populations. For the pre- and post-chemotherapy dataset, GSE222556, METTL3 expression values were extracted from the processed Seurat object, and cells with detectable METTL3 expression (non-zero counts) were retained for downstream stratification. A 70% quantile threshold was applied to the METTL3-positive cells to classify them into METTL3-high and METTL3-low groups. The number and proportion of METTL3-high cells in pre-treatment and post-treatment samples were quantified, and proportional differences between treatment phases were calculated using column-normalized contingency tables. The resulting group proportions were visualized as stacked bar plots to compare the enrichment of METTL3-high cells before and after chemotherapy. Correlation between METTL3 expression and cisplatin sensitivity was evaluated by using the “correlation module” in CPADS. Ovarian serous cystadenocarcinoma (TCGA) was selected as the cancer type, cisplatin as the drug, and METTL3 as the gene of interest. CPADS provides preprocessed METTL3 expression values matched to cisplatin IC50 measurements across TCGA OC samples and performs linear regression and correlation testing automatically. The regression equation, R^2^, and *p*-value reported by CPADS were used directly for analysis and visualization.

### 2.12. Gene-Specific m^6^A RT-qPCR

Purified mRNA (5 μg) was fragmented to an average length of approximately 100 nucleotides by metal-ion-induced hydrolysis. A portion (10%) of the fragmented RNA was reserved as an input control, and the remaining RNA was subjected to m^6^A RNA immunoprecipitation as described above. Gene-specific m^6^A enrichment was quantified by one-step RT–qPCR using Verso SYBR Green reagents (Thermo Fisher Scientific, Waltham, MA, USA) and primers listed in Supplementary Table S1. Enrichment was calculated relative to input RNA using a standard ΔCt/%input-style approach, and m^6^A levels were further normalized to corresponding mRNA abundance when comparing METTL3-overexpressing and control samples.

### 2.13. RNA Stability Assay

mRNA stability was assessed by measuring the rate of transcript decay following transcriptional inhibition. Control-vector- and METTL3-overexpressing cell lines were treated with the transcriptional inhibitor actinomycin D (5 μg/mL) and harvested at 0, 3, and 6 h after treatment. Total RNA was isolated using TRIzol reagent and purified using the RNeasy kit (QIAGEN, Germantown, MD, USA) with on-column DNase I digestion to remove contaminating genomic DNA. Relative ADAM23 mRNA levels were quantified by RT-qPCR using the Bio-Rad iTaq Universal SYBR Green system. The mRNA degradation rate constant (K_decay) was calculated according to the equation ln(C/C_0_) = −K_decay·t, where C_0_ represents the mRNA level at time 0 prior to decay, C represents the mRNA level at time t, and t denotes the duration of transcriptional inhibition. K_decay was derived from exponential decay fitting of C/C_0_ versus time.

The mRNA half-life (t_1_/_2_), defined as the time required for mRNA levels to decrease to 50% of the initial value (C/C_0_ = 1/2), was calculated using the equation ln(1/2) = −K_decay·t_1_/_2_. Rearrangement yields were calculated as t_1_/_2_ = ln(2)/K_decay.

### 2.14. Statistical Analysis

Data are presented as means ± standard deviation (SD). Comparisons between two groups were performed by using the two-tailed Student’s *t*-test, while one-way analysis of variance (ANOVA) was used for comparisons among multiple groups. A *p*-value < 0.05 was considered statistically significant.

## 3. Results

### 3.1. Cisplatin Exposure Is Associated with Increased METTL3 Expression and Global m^6^A Levels in OC Cells

**Figure 1:**
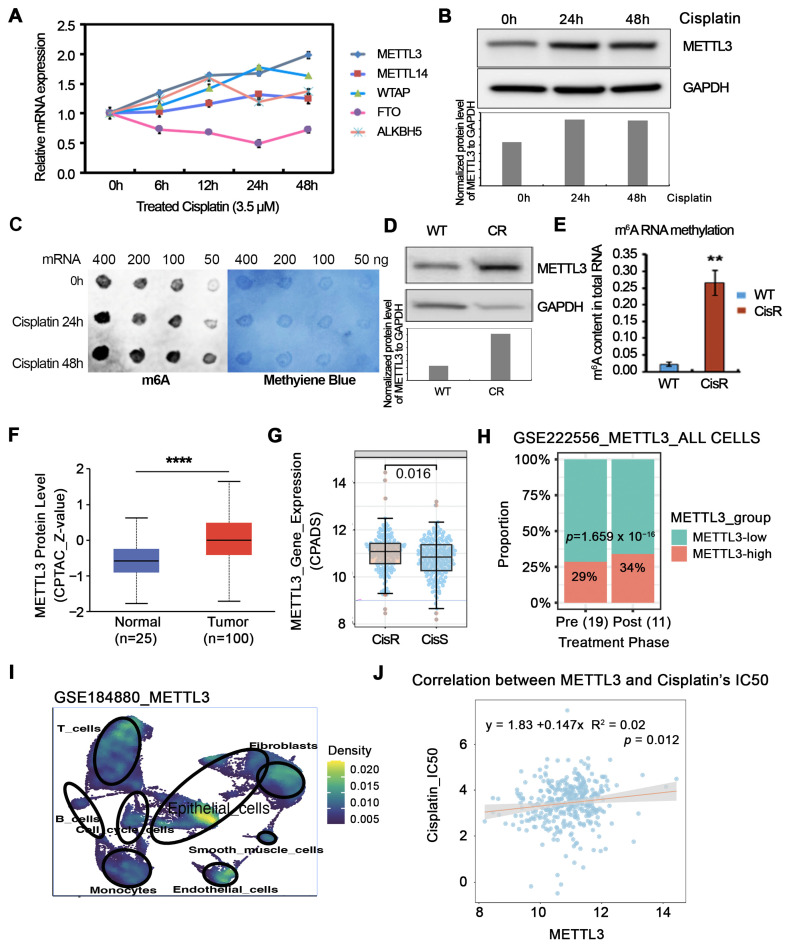
Cisplatin exposure is associated with increased METTL3 expression and global m^6^A RNA methylation. (**A**) mRNA expression levels of RNA-methylation-related genes (METTL3, METTL14, WTAP, FTO, and ALKBH5) in OVCAR5 cells treated with 3.5 µM cisplatin for 0, 6, 12, 24, and 48 h, measured by RT-qPCR. (**B**) Western blot analysis of METTL3 and GAPDH protein expression in OVCAR5 cells treated with 3.5 µM cisplatin for 0, 24, and 48 h. Western blot band intensities were quantified by densitometric analysis using ImageJ (version 1.54g) and normalized to GAPDH. (**C**) Dot blot analysis of m^6^A methylation in OVCAR5 cells treated with cisplatin for 24 and 48 h; methylene blue staining was used as a loading control. (**D**) Western blot analysis of METTL3 and GAPDH protein levels in wild-type (WT, CisS) and CisR OVCAR5 cells. Western blot band intensities were quantified by densitometric analysis using ImageJ and normalized to GAPDH. (**E**) Quantification of global m^6^A methylation levels in wild-type and cisplatin-resistant OVCAR5 cells using the EpiQuik m^6^A RNA methylation quantification kit. Data are presented as means ± SD. ** *p* < 0.01. (**F**) CPTAC proteomics dataset analysis shows METTL3 protein levels in high-grade serous ovarian carcinoma (HGSOC) tumors (*n* = 100) compared with normal ovary (*n* = 25), used as control. Data are presented as means ± SD. **** *p* < 0.0001. (**G**) CPADS transcriptomic analysis shows METTL3 mRNA expression levels in CisR compared to CisS OC specimens from TCGA dataset. (**H**) Single-cell RNA-seq dataset GSE222556: METTL3-high cells (top 30% of METTL3 expression, ≥70th percentile) were more abundant in post-chemotherapy samples (Post) compared to pre-treatment samples (Pre), indicating an enrichment of METTL3-high tumor cell populations following chemotherapy. (**I**) Single-cell RNA-seq dataset GSE184880: Uniform manifold approximation and projection (UMAP) visualization shows enrichment of METTL3 expression within epithelial tumor cell clusters. (**J**) CPADS analysis showing a positive correlation between METTL3 expression and predicted cisplatin IC_50_ values based on transcriptomic signatures across OC samples.

To investigate whether RNA methylation is associated with Pt resistance in OC, we first examined the expression of key m^6^A regulatory components following cisplatin exposure. As shown in Figure 1A, cisplatin treatment was significantly associated with the increased expression of the methyltransferase components *METTL3* and *WTAP*, whereas the RNA demethylase *FTO* was markedly reduced. This transcriptional shift toward enhanced m^6^A deposition was not limited to acute drug exposure. In established cisplatin-resistant (CisR) cells [31,32,33], m^6^A writer genes, including *METTL3*, *METTL14*, and *WTAP*, were consistently upregulated, while the eraser genes *FTO* and *ALKBH5* were significantly downregulated relative to cisplatin-sensitive (CisS) cells (Supplementary Figure S1A). At the protein level, METTL3 expression was also elevated in OC cells following cisplatin treatment compared with untreated controls (Figure 1B). To assess whether these transcriptional and protein-level changes were accompanied by altered RNA methylation, we measured global m^6^A levels following cisplatin exposure. Cisplatin treatment induced a marked increase in total m^6^A levels (Figure 1C and Figure S1B). Similarly, cisplatin-resistant cells exhibited elevated METTL3 protein levels (Figure 1D) together with increased global m^6^A levels compared with cisplatin-sensitive counterparts (Figure 1E). Together, these findings indicate that both acute cisplatin exposure and acquired Pt resistance are associated with increased METTL3 expression and elevated global m^6^A RNA methylation in OC cells.

Next, we investigated the clinical relevance of METTL3 expression in OC using multiple publicly available datasets. Analysis of the CPTAC cohort showed that METTL3 protein levels were significantly higher in OC tissues compared with normal fallopian tube epithelium (Figure 1F). Consistent with this observation, transcriptomic data from the CPADS database revealed that METTL3 mRNA expression was markedly elevated in ovarian tumors categorized as CisR relative to those categorized as CisS (Figure 1G). In addition, at the single-cell level, analysis of the GSE222556 dataset demonstrated that the proportion of METTL3-high cells (cutoff at 70th percentile) was increased in post-chemotherapy samples (34% of all cells) compared with pre-treatment samples (29% of all cells, *p* < 0.0001, Figure 1H). Further interrogation of scRNA-seq data from the GSE184880 dataset showed that METTL3 expression was predominantly enriched within epithelial tumor cell populations relative to stromal and immune cells (Figure 1I). Finally, correlation analysis within the CPADS cohort revealed a positive association between METTL3 expression levels and predicted cisplatin IC_50_ values derived from transcriptomic signatures (Figure 1J), consistent with an association between elevated METTL3 expression and reduced Pt sensitivity.

Overall, our results indicate that cisplatin exposure is associated with increased METTL3 expression and elevated global m^6^A RNA methylation, features that are also enriched in chemoresistant ovarian tumors and cell models. Elevated METTL3 expression correlates with reduced sensitivity to Pt-based therapy, supporting a potential association between METTL3-dependent RNA methylation programs and Pt resistance.

### 3.2. METTL3 Expression Is Associated with Cisplatin Sensitivity in OC Cells

**Figure 2:**
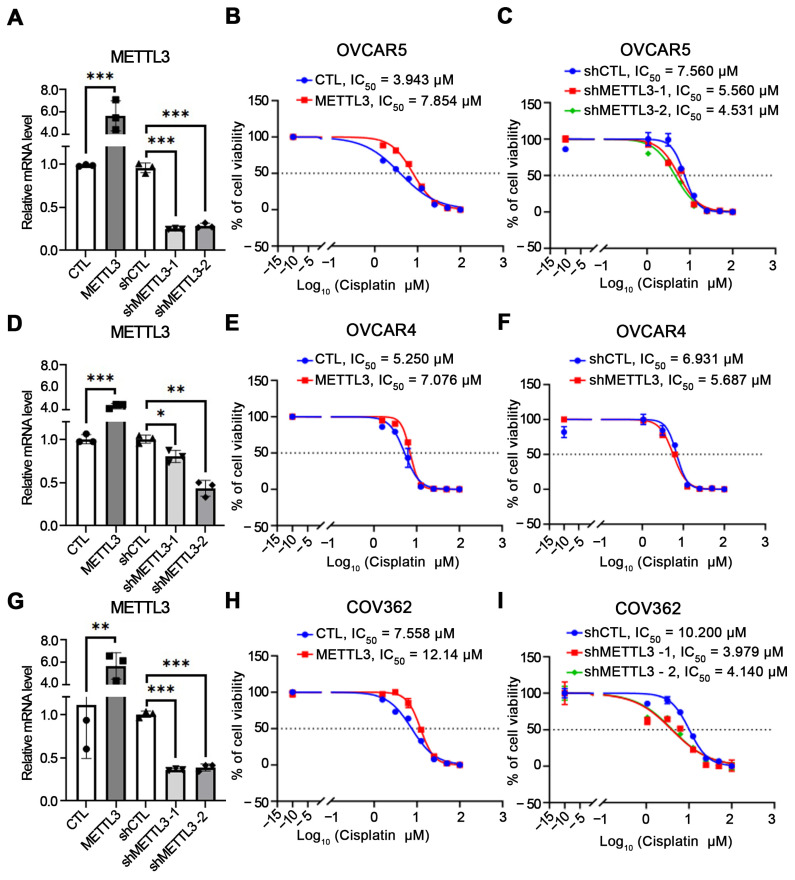
Association between METTL3 expression and cisplatin resistance in OC cells. (**A**) Relative RNA expression of METTL3 in OVCAR5 cells transduced with control vector (CTL) or METTL3-overexpressing construct (METTL3) and in cells with stable knockdown using control shRNA (shCTL) or two independent shRNAs targeting METTL3 (shMETTL3-1, shMETTL3-2). Data are presented as means ± SD. *** *p* < 0.001. (**B**) Cell viability curves of METTL3-overexpressing OVCAR5 cells treated with increasing concentrations of cisplatin. (**C**) Cisplatin sensitivity measured by CCK-8 assay in shCTL, shMETTL3-1, and shMETTL3-2 OVCAR5 cells. (**D**–**F**) Corresponding analyses in OVCAR4 cells: METTL3 expression (**D**), cisplatin response in overexpression model (**E**), and CCK-8 assay in knockdown model (**F**). Data are presented as means ± SD. * *p* < 0.05., ** *p* < 0.01, *** *p* < 0.001. (**G**–**I**) Corresponding analyses in COV362 cells: METTL3 expression (**G**), cisplatin response in overexpression model (**H**), and CCK-8 assay in knockdown model (**I**). Data are presented as means ± SD. ** *p* < 0.01, *** *p* < 0.001. The calculated IC_50_ values for cisplatin are shown for each cell line. Contro groups (PCDH and shCTL) are shown in blue; METTL3 overexpression is shown in red; and METTL3 knockdown is shown using two independent shRNA, shMETTL3-1 (red) and shMETTL3-2 (green).

To examine the relationship between METTL3 expression and cisplatin response, we assessed drug sensitivity in OC cells in which METTL3 levels were genetically manipulated. Forced expression of METTL3 was associated with increased cisplatin IC_50_ values compared with vector-control-transduced cells (Figure 2A,B), indicating reduced sensitivity in the context of elevated METTL3 expression. In contrast, METTL3 depletion using two independent shRNAs (shMETTL3-1 and shMETTL3-2) in OVCAR5 cells was associated with decreased cisplatin IC_50_ (Figure 2A,C). Consistent trends were observed in additional OC cell lines, including OVCAR4 and COV362 cells, in which METTL3 overexpression correlated with higher cisplatin IC_50_ values, whereas METTL3 shRNA-mediated depletion was associated with increased cisplatin sensitivity (Figure 2D–I). These results support a consistent association between METTL3 expression levels and cisplatin response across multiple OC models.

Additionally, a colony-forming assay was used to assess the association between METTL3 expression and OC cell growth. As shown in Supplementary Figure S1C, overexpression of METTL3 was associated with a significant increase in colony number, consistent with enhanced clonogenic growth. Consistent with these observations, correlative survival analysis using publicly available TCGA data for high-grade serous OC from cBioPortal showed that higher METTL3 expression was associated with poorer overall survival (hazard ratio, 1.34; 95 percent confidence interval, 1.16 to 1.55; log rank *P* = 8.6 × 10^−5^; Supplementary Figure S1D).

**Figure 3:**
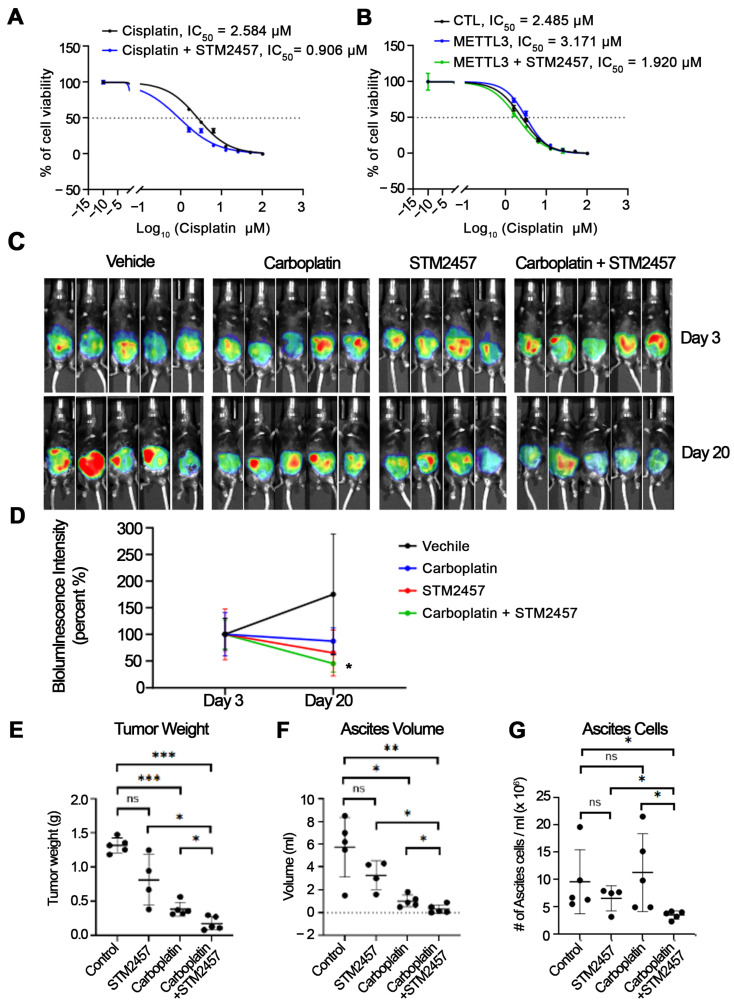
Effects of METTL3 inhibition by STM2457 on Pt sensitivity and tumor burden in vitro and in vivo. (**A**) Cell viability curves of OVCAR5 cells treated with increasing concentrations of cisplatin in the presence or absence of STM2457 (10 µM). Cisplatin-treated cells are shown in black, and cells treated with cisplatin plus STM2457 are shown in blue. (**B**) Cisplatin sensitivity measured by CCK-8 assay in control (CTL), METTL3-overexpressing (METTL3), and METTL3-overexpressing OVCAR5 cells treated with STM2457. CTL is shown in black, METTL3 in blue, and METTL3 plus STM2457 in green. (**C**–**G**) Female C57BL/6 mice were intraperitoneally injected with ID8-luc OC cells and treated with vehicle, carboplatin, STM2457, or a combination of carboplatin + STM2457 (*n* = 5 animals per group). (**C**) Representative IVIS images captured on day 3 (pre-treatment) and day 20 (post-treatment). (**D**) Quantification of bioluminescence intensity before and after drug treatment. Background-corrected flux values were used to assess differences in tumor burden across treatment groups, with bioluminescent intensity normalized to the control group and presented as percent signal relative to baseline. Vehicle is shown in black, carboplatin in blue, STM2457 in red, and carboplatin plus STM2457 in green. (**E**) Total tumor weight at the endpoint. (**F**) Ascites volume measured at the endpoint. (**G**) Total number of cells per mL of ascites (×10^6^) collected at the endpoint. Data are presented as means ± SD. * *p* < 0.05, ** *p* < 0.01, *** *p* < 0.001; ns = not significant.

To further evaluate the functional relevance of METTL3 activity, we utilized STM2457, a selective catalytic inhibitor of METTL3 [24,34]. Treatment with STM2457 was associated with a dose-dependent reduction in global m^6^A RNA methylation, consistent with inhibition of METTL3 enzymatic activity (Supplementary Figure S1E). Pharmacologic METTL3 inhibition was associated with decreased cisplatin IC_50_ values (Figure 3A) and partial attenuation of the METTL3-associated resistant phenotype (Figure 3B). These results support an association between METTL3-dependent RNA methylation and cisplatin responsiveness in OC cells.

We next evaluated the effects of METTL3 inhibition in vivo by using the ID8-luc OC i.p. model, with tumor progression monitored by IVIS bioluminescence imaging. No significant differences in body weight were observed among treatment groups, indicating comparable tolerability across conditions (Supplementary Figure S2A). In contrast, combination treatment with carboplatin and STM2457 was associated with a marked reduction in tumor burden, as assessed by bioluminescence imaging, compared with vehicle control or either carboplatin or STM2457 monotherapy (Figure 3C,D). The combination treatment was also associated with significantly reduced tumor weight and ascites volume at the experimental endpoint relative to control or single-agent treatment groups (Figure 3E,F). To further quantify disease burden, we measured the number of cells present in ascites fluid (cells/mL). Carboplatin alone did not significantly alter ascites cell counts, and STM2457 monotherapy showed only a modest, non-significant trend toward reduction. In contrast, combined treatment with carboplatin and STM2457 was associated with a significant decrease in ascites cell numbers compared with the control and single-agent groups (Figure 3G). To assess the effects of carboplatin and STM2457 in vivo, we measured global m^6^A methylation in RNA extracted from the harvested tumors. Consistent with the findings from in vitro experiments, carboplatin treatment was associated with increased global m^6^A levels in tumors, whereas co-treatment with STM2457 attenuated carboplatin-induced m^6^A RNA methylation (Supplementary Figure S2B).

Collectively, these results indicate that METTL3 activity is associated with Pt resistance in OC. Both genetic depletion and pharmacologic inhibition of METTL3 were associated with increased cisplatin sensitivity in vitro and significantly reduced tumor burden in vivo. Taken together, our findings support a role for METTL3-mediated RNA methylation in modulating chemoresistance and suggest that METTL3 may represent a potential strategy to enhance the efficacy of Pt-based therapy in OC.

### 3.3. Transcriptomic Profiling Identifies METTL3-Dependent Gene Programs Linked to Cisplatin Resistance

**Figure 4:**
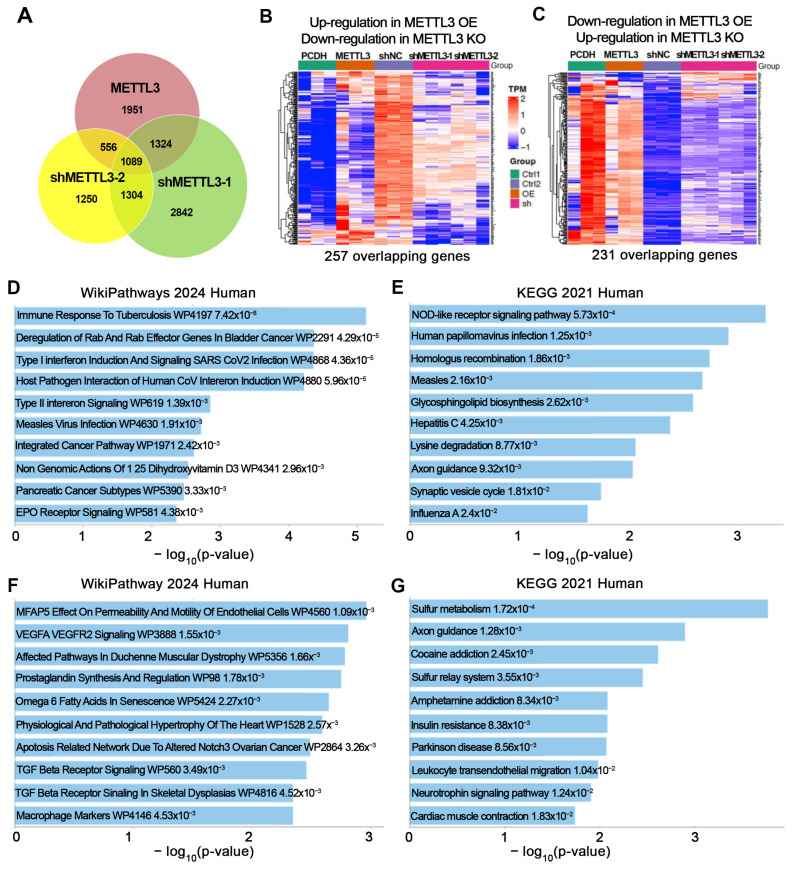
METTL3 is associated with transcriptional programs linked to Pt resistance in OC. (**A**) Venn diagram shows number of overlapping DEGs (FDR < 0.05) identified by RNA-seq in METTL3-overexpressing cells (METTL3) and METTL3 knockdown cells (shMETTL3-1 and shMETTL3-2). (**B**) Heatmap displays significantly upregulated and downregulated genes in METTL3-overexpressing and METTL3-depleted cells (FDR < 0.05). Red indicates higher expression, whereas blue indicates lower expression. Green represents PCDH, orange represents METTL3, Purple represents shNC, and pink represents the two METTL3 shRNA constructs (shMETTL3-1 and shMETTL3-2). (**C**) Heatmap of overlapping DEGs demonstrates distinct clustering between METTL3-overexpressing and METTL3 knockdown groups. Green represents PCDH, orange represents METTL3, Purple represents shNC, and pink represents the two METTL3 shRNA constructs (shMETTL3-1 and shMETTL3-2). (**D**,**E**) Pathway enrichment analysis of genes upregulated by METTL3 overexpression and downregulated by METTL3 knockdown using WikiPathways 2024 Human (**D**) and KEGG 2021 Human (**E**). (**F**,**G**) Pathway enrichment analysis of genes downregulated by METTL3 overexpression and upregulated by METTL3 knockdown using WikiPathways 2024 Human (**F**) and KEGG 2021 Human (**G**).

To explore downstream transcriptional changes associated with METTL3 activity in the context of cisplatin resistance, we performed RNA-sequencing in METTL3-overexpressing and METTL3 KD OC cells, followed by differential expression and pathway analyses. METTL3 overexpression was associated with altered expression of 4920 genes compared with vector control, whereas shMETTL3-1 and shMETTL3-2 KD resulted in 6559 and 4199 differentially expressed genes (DEGs), respectively (FDR < 0.05). Integration of these datasets identified 1089 overlapping genes whose expression patterns were consistently associated with METTL3 perturbation (Figure 4A). Among these genes, 257 were upregulated in METTL3-overexpressing cells and downregulated following METTL3 silencing, while 231 displayed the opposite pattern (FDR < 0.05; fold-change ≥ 2; Figure 4B,C). Heatmap visualization of these transcripts revealed clear separation of METTL3-associated transcriptional signatures across experimental conditions (Figure 4B,C).

To further characterize functional pathways associated with METTL3-regulated transcriptional changes, we performed enrichment analysis of overlapping DEGs that were regulated in opposite directions by METTL3 overexpression and METTL3 KD. Pathway enrichment analysis of genes upregulated in METTL3-overexpressing cells and downregulated following METTL3 depletion, using WikiPathways and KEGG, revealed significant enrichment of pathways related to innate immune and stress–response signaling, including the “NOD-like receptor signaling pathway” and “type I interferon signaling”, as well as pathways involved in homologous-recombination-mediated DNA-damage repair (Figure 4D,E). These pathways have been previously associated with cellular responses to chemotherapy-induced genotoxic stress [6,35].

Conversely, functional analysis of genes downregulated in METTL3-overexpressing cells and upregulated following METTL3 depletion revealed significant enrichment of pathways related to VEGF/VEGFR2 signaling, TGF-β signaling, and Notch3-associated apoptotic regulation (Figure 4F,G). These pathways have been previously implicated in the regulation of cell-adhesion dynamics, tumor microenvironment interactions, and sensitivity to apoptosis, biological processes that are frequently altered during Pt treatment and the development of a resistant phenotype [36,37].

Taken together, these results demonstrate that METTL3 is associated with transcriptional remodeling in OC under Pt-induced stress, characterized by regulation of DNA-repair, cell survival, and apoptotic pathways. This METTL3-associated transcriptional reprogramming may contribute to chemoresistance through coordinated modulation of gene networks involved in cisplatin response.

### 3.4. ADAM23 Is a METTL3-Regulated Transcript Associated with Pt

**Figure 5:**
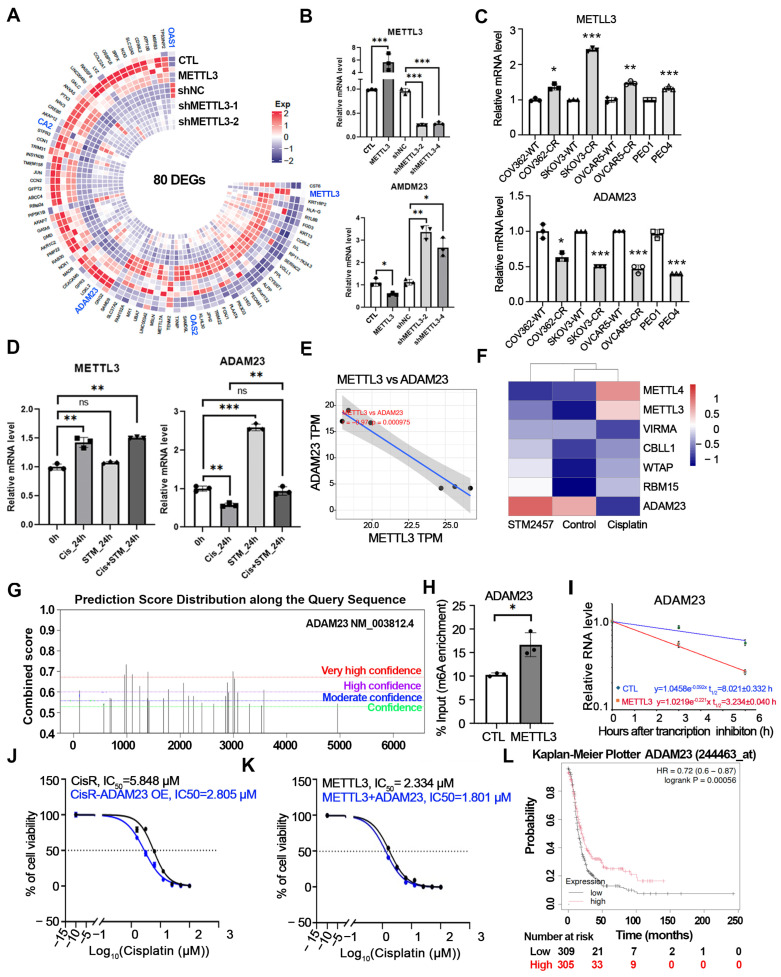
ADAM23 is a METTL3-regulated transcript associated with Pt resistance in OC. (**A**) Heatmap shows the top 80 overlapping DEGs identified from RNA-seq analysis of METTL3-overexpressing and METTL3 knockdown models, highlighting candidates including *ADAM23*, *OAS1*, *OAS2*, *CA2*, and *HLA-G.* (**B**) RT-qPCR validation of ADAM23 expression in METTL3-overexpressing and METTL3 knockdown cells. Data are presented as means ± SD. * *p* < 0.05, ** *p* < 0.01, *** *p* < 0.001. (**C**) RT-qPCR analysis of METTL3 and ADAM23 mRNA expression in CisS (denoted as WT) and CisR OC cell lines, including COV362, SKOV3, OVCAR5, PEO1 (sensitive), and PEO4 (resistant). Data are presented as means ± SD. * *p* < 0.05, ** *p* < 0.01, *** *p* < 0.001. (**D**) RT-qPCR analysis of ADAM23 and METTL3 mRNA levels in control, cisplatin-treated, STM2457-treated, and cisplatin-plus-STM2457-treated OVCAR5 cells. Data are presented as means ± SD. ** *p* < 0.01, *** *p* < 0.001; ns = not significant. (**E**) Correlation analysis of METTL3 and ADAM23 expression based on RNA-seq data from cisplatin-treated OVCAR5 cells. Each dot represents an individual sample. (**F**) Heatmap showing the expression of m^6^A writer complex components (*METTL4*, *METTL3*, *VIRMA*, *CBLL1*, *WTAP*, *RBM15*) and *ADAM23* in STM2457-treated, control, and cisplatin-treated cells. Expression values are scaled by row. Red indicates higher expression, whereas blue indicates lower expression, based on RNA-seq data from cisplatin-treated OVCAR5 cells. (**G**) Predicted m^6^A consensus motifs within ADAM23 mRNA identified by SRAMP web analysis. (green, moderate confidence; blue, high confidence; purple, high confidence; red, very high confidence). (**H**) Gene-specific m^6^A RT-qPCR showing relative m^6^A enrichment on ADAM23 transcripts in control cells (CTL) and METTL3-overexpressing cells (METTL3). Data are presented as means ± SD. * *p* < 0.05. (**I**) Actinomycin D-based mRNA decay analysis of ADAM23 mRNA transcript in control (CTL) and METTL3-overexpressing cells. Red line indicates METTL3 overexpressing cells, whereas blue indicates control cells. (**J**) CCK-8 assay of CisR cells transduced with control vector or ADAM23 and exposed to graded cisplatin concentrations. The calculated cisplatin IC_50_ values for each condition are displayed. Blue line indicates CisR-ADAM23 OE cells, whereas black indicates CisR cells. (**K**) CCK-8 assay of METTL3-overexpressing cells transduced with control vector or ADAM23 and treated with increasing concentrations of cisplatin. The corresponding cisplatin IC_50_ values are shown. Blue indicates METTL3 + ADAM23 cells, and black indicates METTL3-overexpressing control cells. (**L**) Kaplan–Meier analysis of the TCGA OC cohort comparing overall survival between patients with high (red) versus low (black) ADAM23 expression.

To validate the transcriptomic changes induced by METTL3 and to assess the consistency of METTL3-dependent gene regulation, we examined expression patterns of the top eighty overlapping differentially expressed candidates identified through integrative analysis, including *ADAM23*, *OAS1*, *OAS2*, *CA2*, and *HLA-G* (Figure 5A and Supplementary Table S2). As shown in Figure 5B and Figure S3, these transcripts exhibited concordant expression changes across both METTL3 overexpression and METTL3 KD models, consistent with regulation in a METTL3-dependent manner. Among these candidates, *ADAM23* emerged as a consistently regulated transcript, showing marked downregulation in METTL3-overexpressing cells and significant upregulation following METTL3 KD (Figure 5B, bottom panel). ADAM23 encodes an adhesion-related tumor suppressor previously reported to inhibit invasion and metastasis in several malignancies, including breast and pancreatic cancers [20,23,38]. Quantitative RT-qPCR confirmed that ADAM23 expression was reduced in CisR OC cells across multiple models (OVCAR5, SKOV3, COV562, and PEO4 cells) compared with parental sensitive cells (Figure 5C, bottom panel). In contrast, METTL3 expression levels were increased in Pt-R OC cells, inversely correlated with ADAM23 expression (Figure 5C, top panel). We next examined whether cisplatin exposure modulates METTL3-associated regulation of *ADAM23*. Cisplatin treatment was associated with reduced *ADAM23 mRNA* levels accompanied by increased METTL3 expression (Figure 5D). Inhibition of METTL3 catalytic activity using STM2457 did not alter METTL3 transcript levels, as expected, but was associated with a significant increase in ADAM23 mRNA expression (Figure 5D). Notably, STM2457 treatment attenuated cisplatin-associated suppression of *ADAM23*, suggesting that downregulation of *ADAM23* during Pt treatment occurs, at least in part, through a METTL3-dependent mechanism. These findings indicate that METTL3 activation contributes to cisplatin-associated transcriptional remodeling and that ADAM23 is regulated as part of a broader METTL3-dependent gene program linked to Pt resistance.

To further determine whether ADAM23 is regulated in a METTL3-dependent manner and to examine its association with Pt resistance and key regulators of m^6^A methylation, we analyzed transcriptomic profiles of OC cells treated with cisplatin, the METTL3 inhibitor STM2457, and vehicle control using RNA-sequencing. These analyses revealed a consistent relationship linking METTL3 activity, global RNA methylation programs, and ADAM23 expression. As shown in Figure 5E, METTL3 and ADAM23 mRNA levels were strongly inversely correlated (r = −0.97), indicating a robust association between the two transcripts. Cisplatin treatment induced coordinated upregulation of multiple m^6^A writer components, including METTL3, METTL4, VIRMA, CBLL1, and WTAP, consistent with a shift toward a hypermethylated RNA state (Figure 5F). In contrast, METTL3 inhibition by STM2457 was associated with reduced expression levels of writer components, consistent with a hypomethylated RNA signature. ADAM23 expression tracked closely with these methylation states, showing downregulation in cisplatin-treated cells, where writer expression was elevated, and increased expression following STM2457 treatment. This inverse METTL3–ADAM23 relationship was reproduced in both cisplatin-resistant and cisplatin-sensitive OVCAR5 and COV362 cells, supporting the generalizability of this regulatory association (Supplementary Figure S4A,B). Consistent with in vitro observations, carboplatin treatment in vivo was associated with a modest increase in Mettl3 expression and a corresponding decrease in Adam23 expression in tumor RNA from the ID8 intraperitoneal mouse model, although these changes did not reach statistical significance (Supplementary Figure S4C,D). Notably, STM2457 treatment, particularly in combination with carboplatin, was associated with increased Adam23 mRNA expression in ID8 intraperitoneal mouse models (Supplementary Figure S4C,D). Together, these findings support the relevance of the proposed METTL3-associated regulatory relationship involving ADAM23 in both in vitro and in vivo contexts.

To determine whether ADAM23 is regulated in an m^6^A-associated manner, we performed motif prediction analysis using the Sequence-based RNA Adenosine Methylation site Predictor (SRAMP) [39], which identified multiple high-confidence m^6^A consensus motifs within the ADAM23 mRNA transcript (Figure 5G). Consistent with this prediction, gene-specific m^6^A quantitative RT-qPCR following m^6^A immunoprecipitation revealed significantly increased m^6^A enrichment on ADAM23 transcripts in METTL3 overexpressing cells compared with control cells (Figure 5H). To assess whether METTL3 expression is associated with altered ADAM23 mRNA stability, METTL3-overexpressing cells were treated with actinomycin D. The apparent half-life of ADAM23 mRNA was reduced in METTL3-overexpressing (3.234 h) relative to vector-control-transduced cells (8.021 h; Figure 5I). Together, these results support an association between METTL3-dependent m^6^A modification and reduced ADAM23 mRNA stability, consistent with post-transcriptional regulation through this pathway.

To evaluate the functional relevance of ADAM23 in the context of Pt-resistance, we generated ADAM23-overexpressing cells in parental OC lines, CisR cells, and METTL3-overexpressing cells (Supplementary Figure S4E–G). While ADAM23 overexpression did not significantly alter colony-forming capacity (Supplementary Figure S4H), it was associated with reduced spheroid formation, indicating impaired three-dimensional growth (Supplementary Figure S4I). ADAM23 overexpression resulted in only a modest reduction in cisplatin IC_50_ values in CisS cells (Supplementary Figure S4J). In contrast, restoration of ADAM23 expression in CisR cells was associated with a significant increase in cisplatin sensitivity (Figure 5J). Similarly, forced expression of ADAM23 in METTL3-overexpressing OVCAR5 cells was associated with reduced cisplatin IC_50_ values compared with METTL3-overexpressing control cells (Figure 5K), partially reversing the resistant phenotype. Consistent with its reported tumor-suppressive role in other cancer models [22,40], survival analysis of TCGA OC datasets demonstrated that higher ADAM23 expression was associated with improved overall survival (Figure 5L). Collectively, these findings support ADAM23 as a functionally relevant, METTL3-regulated transcript whose reduced expression is associated with Pt resistance. Restoration of ADAM23 expression or pharmacologic inhibition of METTL3-dependent RNA methylation may, therefore, represent a potential therapeutic strategy to enhance Pt responsiveness in OC.

## 4. Discussion

Pt-based chemotherapy remains the cornerstone of frontline treatment for OC; however, the majority of patients eventually relapse with Pt-resistant disease, which remains a critical barrier to improved patient survival [6]. Recent evidence suggests that epitranscriptomic modifications contribute to treatment failure across cancer types [41]. In this study, we identify METTL3-associated m^6^A regulation as an important contributor to the development of cisplatin resistance in OC and describe a previously unrecognized METTL3-ADAM23 regulatory relationship associated with response to Pt.

Our work demonstrates that METTL3 expression and global m^6^A levels are markedly elevated following cisplatin exposure and in Pt-R OC models, consistent with prior reports linking m^6^A methyltransferases to tumor progression and drug tolerance [42,43]. Gain- and loss-of-function studies showed that METTL3 overexpression enhances cisplatin resistance, whereas genetic depletion or pharmacologic inhibition of METTL3 is associated with downregulation of the RNA chemosensitivity. We had previously noted that Pt resistance was associated with downregulation of the RNA demethylase FTO [44]. The results presented here and prior observations highlight that m^6^A modifications and METTL3 activity are closely linked to Pt response and expand our understanding of how RNA modifications contribute to therapy resistance in cancer.

Importantly, pharmacologic inhibition of METTL3 using STM2457-sensitized OC cells on cisplatin in vitro and was associated with reduced tumor burden and ascites formation in vivo in combination with carboplatin, underscoring the translational potential of targeting m^6^A-regulated pathways in Pt-R OC. By identifying a METTL3-dependent, m^6^A-associated regulatory relationship involving ADAM23, our work further illustrates how epitranscriptomic alterations may shape therapeutic vulnerability in OC. Consistent with reports that METTL3 contributes to chemoresistance in other tumor types [43,45], these findings support the involvement of m^6^A-associated mechanisms in Pt resistance in OC. Given the acceptable safety profile of first-generation m^6^A inhibitors [34,46,47] and the observed activity of METTL3 blockade, therapeutic disruption of this pathway may warrant further investigation as a strategy to overcome Pt resistance. Future studies will be essential to define the broader m^6^A landscape in CisR tumors and to identify additional METTL3-regulated transcripts that influence tumor cell survival or immune evasion.

Mechanistically, we identified ADAM23 as a transcript whose expression is associated with METTL3-dependent m^6^A regulation. This is the first evidence linking epitranscriptomic regulation to ADAM23 mRNA stability. This metalloproteinase was characterized as a tumor suppressor [22] and shown to act as a negative regulator of integrin-mediated cell-to-matrix adhesion through direct interaction with the αvβ3 integrin [20]. Although its functional role in Pt response has not been previously delineated, integrin-dependent survival pathways, including FAK, ILK, and AKT signaling, are well-established drivers of cell-adhesion-mediated drug resistance in OC [48,49,50,51]. Integrins such as β1, α5β1, and αvβ3 protect OC cells from cisplatin-induced apoptosis and promote invasive and stem-like phenotypes, and their expression correlates with poor clinical outcomes [52,53,54]. Reduced ADAM23 expression, often associated with promoter hypermethylation, has been linked to metastasis, aggressive tumor biology, and decreased survival in OC [55].

Given the established effects of ADAM23 in integrin signaling, reduced ADAM23 expression in METTL3-high settings provides a biologically coherent explanation for how m^6^A-associated regulation may contribute to Pt resistance. Based on the robust involvement of integrin signaling in the response to Pt [56,57], restoration of ADAM23 function could represent a potential strategy to enhance sensitivity to cisplatin and carboplatin. By reducing αvβ3 and β1 integrin activation, ADAM23 may weaken extracellular matrix-dependent survival programs and thereby increase susceptibility to Pt-induced apoptosis.

Our findings further indicate that Pt exposure is associated with increased METTL3 expression and global m^6^A levels, accompanied by reduced ADAM23 mRNA stability. While our data establish a functional association between METTL3-dependent m^6^A regulation and ADAM23 repression, the specific m^6^A reader proteins mediating this process were not examined and remain an important direction for future investigation. Nevertheless, METTL3-associated modulation of ADAM23 expression in Pt-treated OC models underscores the functional relevance of this regulatory axis in shaping treatment response. Together, these results highlight ADAM23 as a functionally relevant METTL3-regulated contributor whose restoration may help counteract Pt resistance and enhance the efficacy of Pt-based chemotherapy in OC.

## 5. Conclusions

In summary, these findings identify a previously unrecognized epitranscriptomic mechanism of Pt resistance in OC involving METTL3-dependent m^6^A regulation of ADAM23. The METTL3–ADAM23 regulatory relationship highlights a potential vulnerability in Pt-resistant OC and suggests that inhibition of this m^6^A-regulated pathway may enhance drug sensitivity. More broadly, our data emphasize the importance of RNA-methylation-associated regulatory programs in shaping therapeutic response and support further investigation of epitranscriptomic targets as a strategy to improve treatment outcomes for OC patients.

## Data Availability

The data generated in this study are available in the Gene Expression Omnibus (GEO). RNA-seq data comparing METTL3 overexpression versus PCDH control and shMETTL3-1 and shMETTL3-2 versus shNC are deposited under accession numbers GSE313201 and GSE313200, respectively. Single-cell RNA-sequencing datasets analyzed in this study were obtained from publicly available resources in the GEO database under accession numbers GSE184880 and GSE222556.

## Supplementary Materials

The following supporting information can be downloaded at https://www.mdpi.com/article/10.3390/cells15030294/s1: Table S1: List of primers oligonucleotides. Table S2: List of top 80 overlapping significantly differentially expressed genes in RNA-seq, including METTL3 vs. CTL, shMETTL3-1 vs. sh-control, and shMETTL3-2 vs. sh-control. Figure S1: METTL3 expression. Figure S2: Body weight monitoring and global RNA methylation changes following carboplatin and STM2457 treatment. Figure S3: METTL3 regulates expression of interferon response genes. Figure S4: ADAM23 expression in cisplatin-resistant cells and functional impact of ADAM23 overexpression.
