# Supplementary material for: Epitranscriptomic Regulation of Platinum Resistance via the METTL3-ADAM23 Axis in Ovarian Cancer
Source: Cells. 2026 Feb 4;15(3):294. doi: 10.3390/cells15030294 (PMC12896498; doi:10.3390/cells15030294)
Supplement: Supplementary file 1 [file cells-15-00294-s001.zip › cells-4074953-supplementary.pdf]

Supplemental table S1. List of primers oligonucleotides

Target gene	Forward primer (5' to3')	Reverse primer (5' to 3')	length
18S	GAGACTCTGGCATGCTAACTAG	GGACATCTAAGGGCATCACAG	129
METTL3	CAAGGAGGAGTGCATGAAAG	GGAAAGAGCAGTCACCTAAAG	110
METTL14	TTTCATCAGGGATGTAGGTTTAG	CATGGGAGGAGTGTTAGATTTAG	101
WTAP	CTACTCAAATCCAGTACCTCAAG	CTGTTCCAGTTCACCTTTCA	119
FTO	TGCCAAAGAGAAGGCTAATG	GTATGCTGCTCTGCTCTTAAT	124
ALKBH5	AGTTCCAGTTCAAGCCTATTC	CCGTATGCAGTGAGTGATTT	106
ADAM23	AATACAGAAGGCACTGAGAAG	GTGGAGCTCGAGTAAGATTG	118
CA2	CAAGAGTGCTGACTTCACTAAC	CAATCCAGGTCACACATTCC	119
HLA-G	CCTACGATGGCAAGGATTAC	GTAGGCTCTCCTTTGTTCAG	128
OAS1	GTTGACTGGCGGCTATAAA	TCAGGAAGTCTCTCTGTAGTT	122
OAS2	CAACGTGACATCCTCGATAAA	CAGCACCTCGAAAGAGATTC	150
Mus-Mettl3	GTCAGGAGATCCTAGAGCTATTA	CTCTTCCTTGGTCCCATAATC	122
Mus-Adam23	TTCGGCTCCAAGTTCATTC	CTCTCCACCCTTAGAGTACAT	111
ADAM23-m6A	CATCAGAGGCGTCAAAGAC	CAGCTCTAGTGGCTCTATCA	106

Supplemental table S2. List of top 80 overlapping significant differentially expressed genes in RNA-seq including METTL3 vs CTL, shMETTL3-1 vs shRNA control, and shMETTL3-2 vs shRNA control

**Symbol**	**log2FC(METTL3/CTL)**	**file1_pvalue**	**file1_padj**	**log2FC(shMETTL3-1/shNC)**	**file2_pvalue**	**file2_padj**	**log2FC(shMETTL3-2/shNC)**	**file3_pvalue**	**file3_padj**
CST6	1.37	1.84E-75	3.77E-72	-0.26	3.01E-07	3.83E-06	-0.65	1.88E-23	4.42E-21
METTL3	1.29	2.32E-96	2.14E-92	-0.75	7.38E-46	1.45E-43	-0.63	2.58E-23	5.98E-21
KRT16P2	1.18	0.00952	0.0562	-0.99	0.0008974	0.00530022	-0.77	0.00748456	0.05169647
HLA-G	1.18	0.00436	0.0304	-0.36	0.03833989	0.1233106	-0.48	0.00647283	0.04622445
RTL8B	1.16	1.30E-05	0.000202	-0.73	0.00011376	0.00084633	-0.40	0.04683166	0.18939331
FGD3	1.07	9.71E-13	5.19E-11	-0.19	0.01124507	0.04577412	-0.43	2.69E-08	1.02E-06
KRT13	1.05	1.76E-07	4.13E-06	-2.00	1.13E-106	9.87E-104	-0.33	3.02E-05	0.00055746
CCRL2	0.95	6.93E-06	0.000115	-0.35	0.01967592	0.07198884	-0.30	0.04684043	0.18939331
IVL	0.90	0.00637	0.0411	-0.69	9.99E-08	1.39E-06	-0.91	5.02E-11	2.96E-09
RP11-7K24.3	0.90	3.37E-06	6.04E-05	-0.43	8.08E-05	0.00062147	-0.51	3.31E-05	0.0006021
SERINC2	0.89	8.09E-55	7.12E-52	-0.26	2.73E-17	1.09E-15	-0.30	4.08E-11	2.43E-09
VGLL1	0.88	4.27E-07	9.44E-06	-1.58	2.84E-64	1.05E-61	-0.56	2.94E-10	1.58E-08
PPL	0.88	1.13E-25	2.12E-23	-0.44	3.02E-48	6.38E-46	-0.31	1.51E-07	4.76E-06
CYSRT1	0.88	2.36E-13	1.37E-11	-0.28	6.84E-05	0.00053356	-0.29	0.0001645	0.00235199
ALPP	0.86	0.00015924	0.00186955	-0.40	0.01749632	0.06552503	-0.82	7.29E-06	0.00015905
C6orf132	0.84	2.71E-29	6.60E-27	-0.18	2.01E-07	2.66E-06	-0.24	0.00035126	0.00448235
PECAM1	0.70	1.08E-05	0.0001722	-1.32	1.12E-34	1.33E-32	-0.74	3.69E-11	2.22E-09
LY6D	0.65	4.48E-08	1.17E-06	-1.60	5.22E-223	2.01E-219	-0.73	4.57E-34	2.19E-31

PIK3CG	0.65	0.00013466	0.00161585	-0.83	1.34E-10	2.77E-09	-0.80	7.97E-08	2.65E-06
PLAAT4	0.56	6.32E-15	4.47E-13	-0.89	3.79E-103	3.04E-100	-0.22	0.00033102	0.00425953
FOXJ1	0.54	0.00289552	0.02180371	-2.85	1.03E-135	1.41E-132	-1.20	1.47E-35	7.76E-33
TRIM22	0.51	0.02831253	0.12934169	-1.50	7.85E-22	4.38E-20	-0.94	3.50E-11	2.12E-09
JPH2	0.46	0.00098853	0.00891	-1.00	1.18E-26	9.13E-25	-0.46	3.15E-06	7.56E-05
KLHL30	0.45	0.02279411	0.109	-0.85	9.33E-06	8.75E-05	-0.66	0.00194289	0.0179118
OAS2	0.43	0.03713023	0.0157	-1.20	9.84E-14	2.91E-12	-1.03	2.88E-11	1.76E-09
SAMD9L	0.42	0.00025243	0.00279152	-1.03	3.76E-58	1.15E-55	-0.74	1.77E-23	4.23E-21
TXNIP	0.38	4.11E-11	1.72E-09	-1.12	2.92E-241	2.81E-237	-0.36	2.49E-14	2.43E-12
TENM2	0.34	0.03328591	0.14561797	-1.51	0.03568253	0.11618455	-1.57	0.03009976	NA
METTL7A	0.34	0.03865747	0.16224195	-0.98	3.50E-10	6.88E-09	-0.37	0.01348362	0.08037162
MSLN	0.34	0.00011032	0.00136089	-1.48	1.44E-146	2.31E-143	-0.70	1.41E-25	4.57E-23
LINC00342	0.31	0.01350024	0.0338954	-0.88	5.39E-12	1.33E-10	-0.48	0.00151972	0.01471016
UBA7	0.24	0.02436064	0.0115	-0.84	3.43E-23	2.10E-21	-0.18	0.04100126	0.17333993
MX1	0.21	0.02727011	0.0126	-1.33	3.10E-115	3.13E-112	-1.30	3.98E-68	6.10E-65
FAM102A	0.20	0.00427234	0.02990474	-0.83	1.17E-78	5.74E-76	-0.25	2.16E-05	0.00041839
SLC37A2	0.52	2.65E-12	1.33E-10	-0.68	4.66E-32	4.71E-30	-0.88	8.17E-44	8.34E-41
SAMD9	0.36	1.57E-05	0.00023989	-0.73	1.19E-57	3.48E-55	-0.88	9.59E-31	4.08E-28
GNG2	-0.79	1.94E-13	7.38E-46	1.37	8.87E-15	2.86E-13	0.65	0.00174493	0.01636258
ADAM23	-0.71	1.06E-12	6.57E-19	1.37	1.37E-14	4.33E-13	0.80	6.40E-05	0.00106922
LOXL2	-0.68	7.52E-34	6.76E-22	1.89	5.74E-234	3.67E-230	1.34	5.21E-83	9.96E-80
GPR3	-0.63	1.77E-05	0.000768	1.10	3.47E-10	6.84E-09	0.64	0.00130816	0.01310983
CEACAM5	-0.59	1.49E-16	3.16E-0.5	1.51	3.70E-193	1.18E-189	0.65	2.45E-24	6.37E-22
MAOB	-0.58	0.00822898	3.75E-121	0.84	0.00359507	0.01741833	0.76	0.01402273	0.08281143
NOX1	-0.58	0.02809326	3.01E-07	1.12	6.14E-07	7.29E-06	0.67	0.00888772	0.05866279
RAB30	-0.55	4.76E-06	1.65E-17	1.02	8.96E-11	1.90E-09	0.68	9.26E-05	0.0014505
PMP22	-0.55	3.48E-08	8.13E-52	0.83	7.41E-07	8.60E-06	1.21	3.64E-11	2.19E-09
AKR1C2	-0.53	1.44E-05	4.40E-20	1.25	6.68E-19	3.00E-17	0.41	0.02794843	0.13462544
DMD	-0.52	0.00011193	2.58E-54	0.82	5.15E-08	7.50E-07	0.59	0.0002711	0.00358857
GATA6	-0.52	1.62E-06	4.19E-34	0.92	4.76E-15	1.56E-13	0.40	0.00398153	0.03183769
AKAP7	-0.51	0.00590669	4.14E-10	0.88	0.00014667	0.00105557	0.78	0.00102115	0.01079897
PIP5K1B	-0.47	0.03532458	0.0248	1.20	2.32E-13	6.58E-12	0.99	1.89E-08	7.32E-07
RBM24	-0.45	3.24E-05	1.25E-06	1.04	6.08E-05	0.00048137	1.34	1.36E-07	4.31E-06
ABCC4	-0.44	7.64E-11	4.92E-07	0.86	1.58E-33	1.77E-31	0.40	2.21E-05	0.00042589

GFPT2	-0.44	1.33E-11	2.73E-17	1.32	2.97E-30	2.77E-28	1.46	5.20E-36	2.84E-33
CCN2	-0.43	8.79E-13	2.39E-09	0.82	7.04E-19	3.15E-17	0.59	9.99E-08	3.26E-06
JUN	-0.40	5.07E-09	1.31E-18	0.85	1.73E-33	1.89E-31	0.58	1.98E-13	1.68E-11
TMEM158	-0.34	0.00174452	0.00188	0.81	2.10E-12	5.39E-11	0.60	1.76E-06	4.46E-05
INSYN2B	-0.33	2.26E-06	9.58E-28	1.21	2.68E-32	2.73E-30	0.52	1.01E-05	0.00021175
TRIM31	-0.32	0.00115695	1.72E-27	0.81	1.24E-19	5.86E-18	0.43	3.23E-05	0.00059092
CCN1	-0.31	8.99E-09	1.05E-10	0.84	4.36E-80	2.26E-77	0.47	2.05E-17	2.73E-15
S1PR3	-0.28	0.03191967	2.24E-08	0.92	3.23E-07	4.09E-06	0.98	7.13E-08	2.40E-06
CA2	-0.27	0.00093041	2.95E-05	0.96	1.69E-49	3.78E-47	0.60	1.06E-15	1.15E-13
AKAP12	-0.23	0.00083612	8.14E-07	1.12	5.52E-100	3.92E-97	0.40	8.32E-08	2.74E-06
CREB5	-0.23	0.00521466	9.38E-11	1.26	8.31E-14	2.47E-12	1.22	7.77E-12	5.20E-10
NAV3	-0.22	0.01849498	2.69E-05	1.45	2.71E-24	1.82E-22	0.90	2.47E-08	9.42E-07
PTX3	-0.19	0.00176268	0.0173	0.84	8.55E-31	8.13E-29	0.76	6.24E-22	1.33E-19
ANXA5	-0.16	0.00413865	6.03E-36	0.91	1.27E-117	1.36E-114	0.76	1.64E-51	2.10E-48
GALC	-0.14	0.04372793	7.65E-07	0.85	1.89E-62	6.85E-60	0.30	2.07E-05	0.00040304
LINC00460	-1.16	1.17E-22	1.76E-20	0.71	8.49E-05	0.00065049	0.44	0.01843268	0.10030548
RASSF8	-1.08	2.82E-58	2.61E-55	0.33	4.92E-07	5.96E-06	0.34	0.00010176	0.00157715
LYZ	-0.92	4.77E-33	1.84E-30	0.17	0.00609611	0.02731016	0.66	1.84E-24	5.02E-22
COL22A1	-0.90	4.05E-12	1.98E-10	0.57	0.00819784	0.0351119	1.06	1.14E-07	3.69E-06
OSBPL6	-0.86	1.66E-16	1.45E-14	0.31	0.04884347	0.14893088	0.36	0.03467512	0.15511697
SRPX	-0.81	2.15E-18	2.23E-16	0.62	3.57E-07	4.47E-06	0.54	4.25E-05	0.00075026
NOG	-0.74	3.95E-11	1.66E-09	0.57	0.01416428	0.05546977	1.59	1.20E-14	1.20E-12
SLC22A3	-0.69	1.77E-22	2.58E-20	0.40	0.0048348	0.02245597	1.04	1.74E-14	1.72E-12
CD99L2	-0.54	2.28E-13	1.33E-11	0.19	0.02641824	0.09123028	0.89	7.33E-26	2.44E-23
ATP10B	-0.52	7.36E-07	1.53E-05	0.68	2.80E-19	1.29E-17	0.82	4.88E-24	1.20E-21
MSRB3	-0.49	7.90E-11	3.19E-09	0.76	2.13E-08	3.24E-07	1.16	3.99E-17	5.01E-15
TP53INP2	-0.35	0.00064192	0.00624314	0.43	0.00037485	0.00244565	0.89	4.80E-14	4.37E-12
OAS1	0.27	0.00108985	0.00971036	-0.65	9.90E-30	9.05E-28	-0.64	1.94E-22	4.37E-20

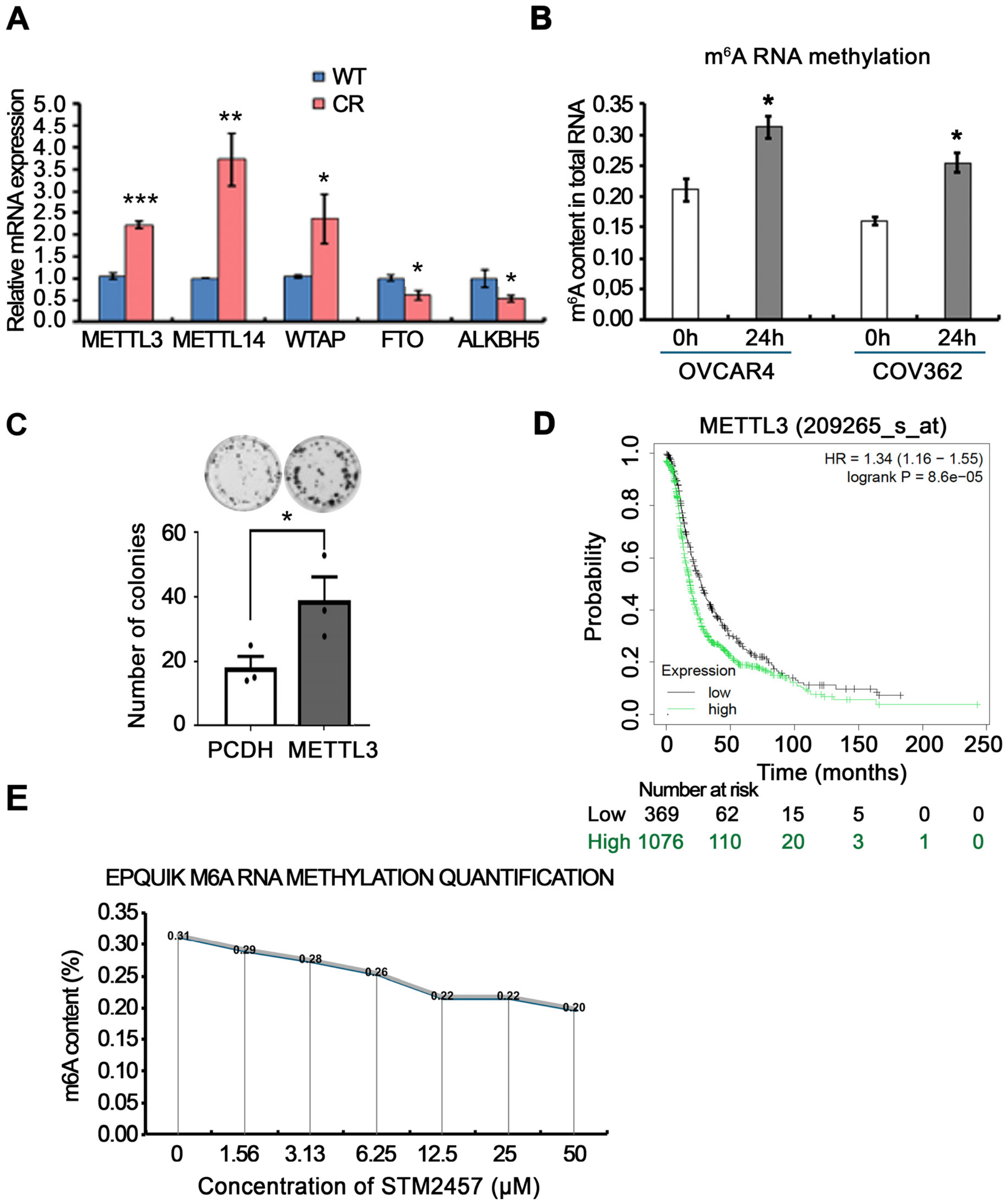

Supplemental Figure S1. METTL3 expression, functional impact, inhibition, and clinical relevance in ovarian cancer.

(A) Relative messenger RNA expression of m^6A regulatory genes in parental (wild type WT) and cisplatin resistant (CR) ovarian cancer cells. CR cells showed marked upregulation of METTL3, METTL14, and WTAP, with modest reductions in FTO and ALKBH5 expression levels. Data represent means +/- standard deviation. Statistical significance was determined using a two tailed Student t test (*P < 0.05, **P < 0.01, ***P < 0.001). (B) Quantification of global m6A methylation levels in wild-type and cisplatin-resistant OVCAR4 and COV362 cells using the EpiQuik m6A RNA methylation quantification kit. (C) Colony formation assay measures numbers of colonies in cells overexpressing METTL3 compared with cells transfected with vector control (PCDH). Representative colony images (top) and quantification (bottom; *P < 0.05) are shown. (D) Kaplan Meier analysis of overall survival using TCGA ovarian cancer database in cBioportal, in patients whose tumors express high (green) vs low (black) METTL3 expression. Numbers at risk for high and low expression cohorts are shown below the curve. (E) Global m6A RNA methylation content following treatment with the METTL3 inhibitor STM2457.

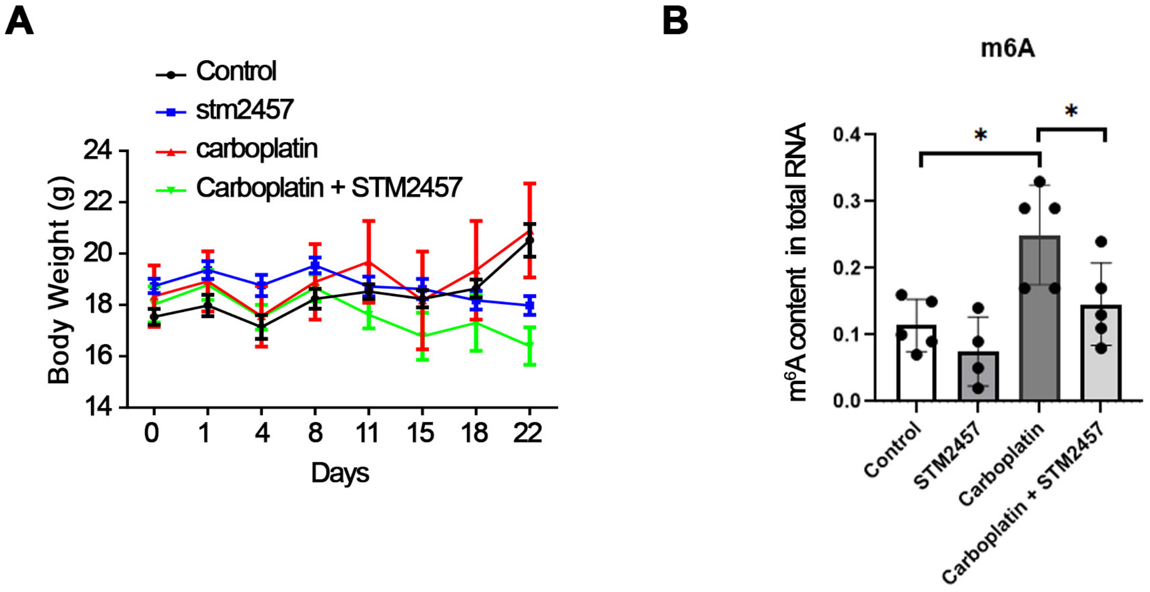

Supplemental Figure S2. Body weight monitoring and global RNA methylation changes following carboplatin and STM2457 treatment.

(A) Body weight of female C57BL/6 mice intraperitoneally injected with ID8-luc ovarian cancer cells and treated with vehicle (control), STM2457, carboplatin, or the combination of carboplatin and STM2457 (n = 5 mice per group). Body weight was monitored at the indicated time points during treatment. Data are shown as means ± SEM. (B) Global m^6A RNA content in xenografts collected at the experimental endpoint. Data are presented as mean ± SEM. *$p < 0.05$.

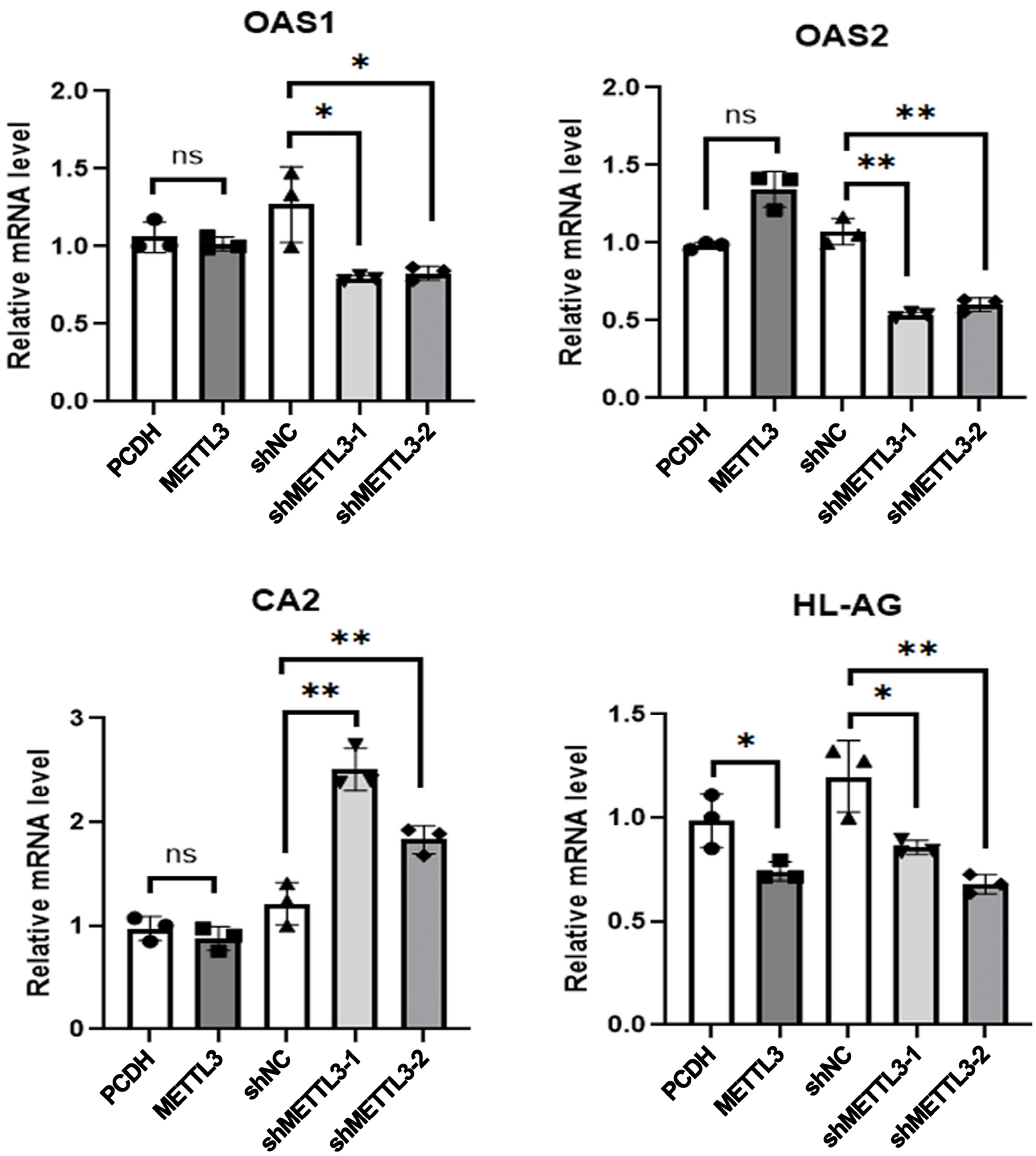

Supplemental Figure S3. METTL3 regulates expression of interferon response genes.

Quantitative RT-PCR assessed relative mRNA levels of selected METTL3 target genes (OAS1, OAS2, CA2, and HL-AG) in OVCAR5 cells transfected with METTL3 (METTL3), control vector (PCDH), non-targeting shRNA (shNC), or METTL3 knockdown using two independent shRNAs

(shMETTL3-1 and shMETTL3-2). Statistical comparisons were performed using a two tailed Student t test (*$P < 0.05$, **$P < 0.01$, ns not significant).

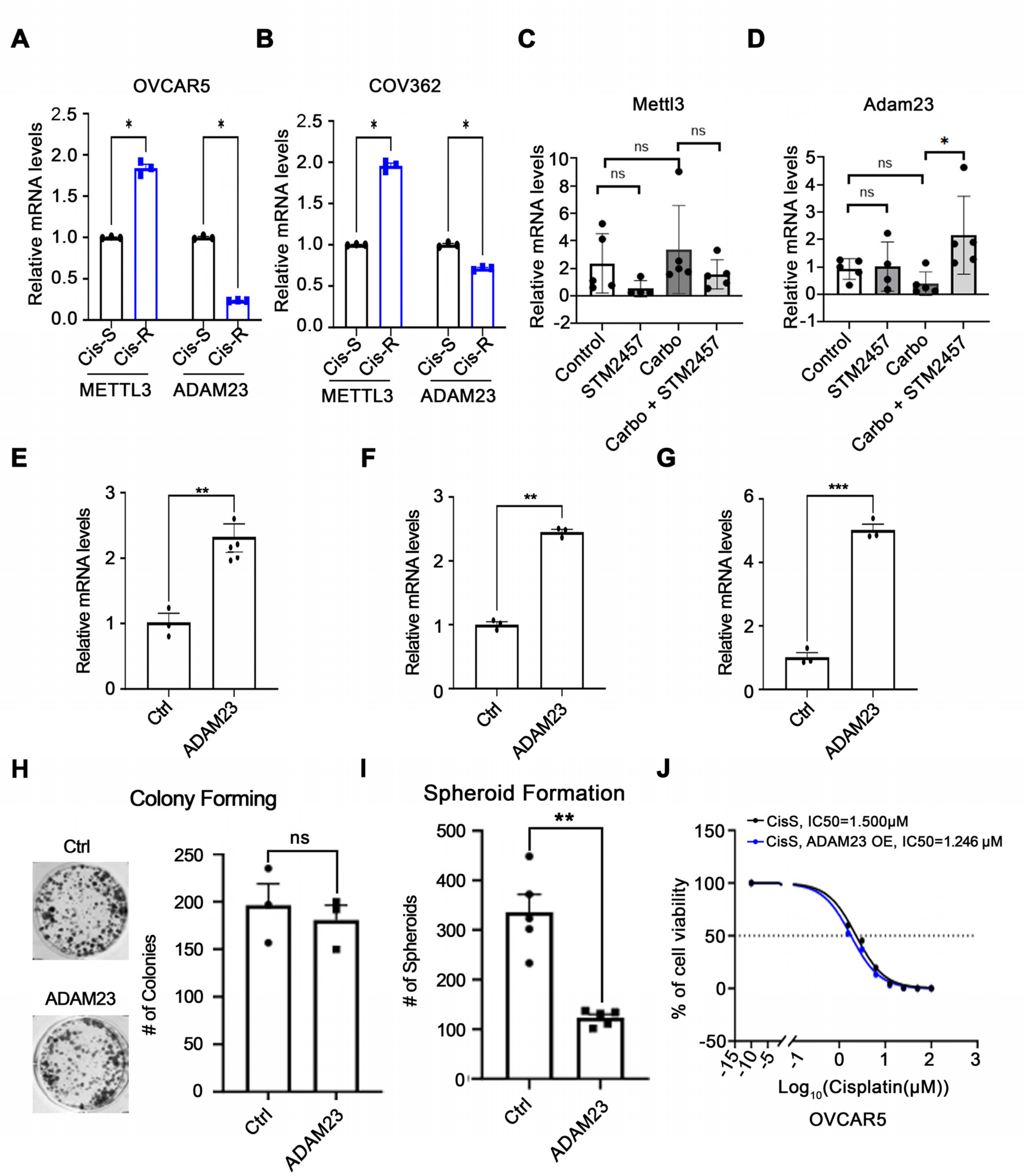

Supplemental Figure S4. ADAM23 expression in cisplatin resistant cells and functional impact of ADAM23 overexpression.

(A) RT-qPCR measures mRNA levels of METTL3 and ADAM23 in CisS and CisR OVCAR5 cells. (*P < 0.05). (B) RT-qPCR measures mRNA expression of METTL3 and ADAM23 in CisS and

CisR COV362 cells. (*P < 0.05). (C) Relative mRNA expression levels of Mettl3 were measured by RT–qPCR in tumor samples collected from ID8-luc–bearing mice treated with vehicle (Control), STM2457, carboplatin (Carbo), or the combination of carboplatin and STM2457. Data are shown as mean ± SEM, with individual data points representing biological replicates. (*P < 0.05; ns, not significant). (D) Relative mRNA expression levels of Adam23 were measured by RT–qPCR in tumor samples collected from ID8-luc–bearing mice treated with vehicle (Control), STM2457, carboplatin (Carbo), or the combination of carboplatin and STM2457. Data are shown as means ± SEM, with individual data points representing biological replicates. (*P < 0.05; ns, not significant). (E–G) RT-qPCR measures ADAM23 expression levels in OVCAR5 parental (WT) cells (E), CisR OVCAR5 cells (F), and METTL3 overexpressing cells (G). (**P < 0.01, ***P < 0.001). (H) Colony formation assay comparing control and ADAM23 overexpressing cells. Representative colony images are shown on the left, with quantification on the right. (ns, not significant). (I) Spheroid formation assay in control versus ADAM23 overexpressing cells. (**P < 0.01). (J) A cell viability CCK8 assay shows responses expressed as IC50 values of OVCAR5 cells transduced with control vector and ADAM23 to cisplatin.
